# De L’identité Du Typhus Et De La Fièvre Typhoïde

**Published:** 1845-03

**Authors:** 


### BIBLIOGRAPHICAL NOTICES.

*De l'Identité du Typhus et de la Fièvre Typhoïde;* par C. E. S. Gaultier de Claubry, Chevalier de l'Ordre Royal de la Légion d l'Honneur, Docteur en Médecine et Agrégé libre de la Faculté de Paris, &c. &c. 8vo. pp. 496. Paris, 1844.

*On the Identity of Typhus and Typhoid Eever.* By C. E. S. Gaultier de Claubry, &c. &c.

The doctrine of the distinct characters of Typhus and Typhoid Fever was promulged a few years ago with more zeal, and we had almost said intolerance, than was discreet or judicious. As in the case of Broussaism, sides were taken by the great contending parties, and the controversy exhibited more of the character of a political than a scientific warfare. Points were considered established that are now abandoned; and scientific doubt has now taken the place of categorical assertion. And who is there now, of all the warm enthusiastic Broussaists, who were so obstreperous not many years ago, that ever refers to views once so warmly cherished, but of which subsequent observation and reflection have shown them the fallacy?

On the subject of typhus and typhoid fevers things are not as they were; and much discordance exists, even among those who believe in their separate and distinct character. Some are of opinion, that the intestinal lesion constitutes the essence of the affection; whilst others regard it as a mere expression of the constitutional malady,—as the variolous eruption is of small-pox, and the morbillous of measles. Once, a grand distinction between typhoid and typhus fever was considered to be—that the former is not communicable, whilst the latter is; and this essential difference was forcibly urged by one of our own writers—Dr. Gerhard—who had no doubt whatever on the matter;—his convictions being based on actual experience in the Philadelphia Hospital. Such, too, *were* the views of M. Louis. *Now*, however, he affirms, that the contagious nature of the typhoid affection is fully demonstrated. M. Bretonneau, of Tours, the great originator of the doctrine as to the intestinal character of this affection, and of the term *Dothinentérite* applied to it, affirms, that he has seen the disease pass from one village to another previously free from it, after the arrival in the latter of one who had remained for a longer or shorter period in the former; and the observations of Gendrin, Putegnat, and others, have so strongly confirmed those of M. Bretonneau, that M. Louis has fully embraced the doctrine of contagion as applicable to typhoid fever. Whether all his disciples have followed, or will follow, the master, does not appear, for many of them are silent; and it will not be easy for them, we apprehend, to bring forward stronger facts in favour of its contagious character than those they adduced to show, that whilst typhus was positively communicable, there was no example of the typhoid affection extending in the same manner.

Again, not many years ago, it was a matter of universal belief, that typhoid fever does not occur in infancy. *Now*, it is shown to be very common amongst children; and valuable information on this head is contained in the excellent work of MM. Rilliet and Barthez. We are told, again, that in Paris it is limited to persons under fifty; yet further observation may exhibit, that there is no more truth in this than in some of the other assertions, that were considered established until farther observation demonstrated their inaccuracy. The same disease in different years and seasons, by no means assumes precisely the same characters. Circumstances, too, may arise that may give occasion to its attacking those of one age at one time, and of another at another; and, consequently, as on every subject of pathological investigation, we must record the observations, not of one or two years or seasons, but of a great number. Such a course has enabled us to establish, that, in the same country, as in England, where typhus generally prevails, but few cases, at times, in the mass, present the affection of the patches of Peyer, which is esteemed characteristic of typhoid fever; at others, almost all the cases exhibit the intestinal lesion; and yet the general ataxic and adynamic phenomena may be the same. These and other facts, which we have not space to detail, have led us to regard the two diseases as but varieties of adynamic fever having different phenomena or expressions under different circumstances; and such appears to be the view of the author of the work before us.

In the year 1835, the Académie Royale de Médecine proposed for the subject of a prize essay, to be determined in 1837—"to point out the analogies and difference between typhus and typhoid fever in the present state of science." For this M. de Claubry contended; and for his Essay, the first *prix d'encouragement* was decreed; and it was, moreover, printed entire in the Collection of Memoirs of the Academy, (vol. vii. p. 1.) This memoir is in reality the basis of his present volume.

In it, M. de Claubry attempted to show, that the typhoid fever of M. Louis is the same as the typhus of armies, with which he, from his long military service was familiar. This, he thinks, he then demonstrated thoroughly; and others, amongst whom are Messrs. Chomel and Louis, frankly admit it; the former stating, that the establishment of such identity settles the question in regard to the contagious nature of typhoid fever in the affirmative; whilst the latter, in the 2d edition of his *Recherches sur la fièvre typhoïde*, (vol. 2, p. 311,) thus expresses himself:—"L'étude comparée des symptomes indique l'identité des deux maladies." ("The comparative study of the symptoms indicates the *identity* of the two diseases;") a pregnant sentence, which M. de Claubry has placed on the title page of his present production.

As regards, then, the identity of "camp fever" or "army typhus," and the typhoid affection of Paris, there seems to be no difference of sentiment between M. de Claubry and the great Parisian authorities on the latter affection; and this unanimity would seem to have been mainly brought about by the arguments of M. de Claubry in the memoir in question, and of which we stated, that the present work is a *rifacimento*, with additional reflections suggested by subsequent experience.

We cannot follow the author in his elaborate inquiry into the comparative symptomatology, as deduced from different epidemics of typhoid and typhus affections, the former obtained chiefly from the writings of French army surgeons,—for M. de Claubry does not appear to know much of what has been done, or is doing, elsewhere,—in this respect resembling too many of his scientific countrymen,—nor into the pathological anatomy, causes, treatment and prophylaxis. We have only space for his conclusions, which are,—that "under the quadruple relation of pathogenic condition, characteristic symptoms, anatomical alterations, and specific contagious character of the affections, *typhus* and *typhoid fever* are one and the same malady,—a *specific contagious exanthematous fever*:"—and he concludes: "*Typhus* and *Typhoid Fever* are one and the same malady, to which it will be well to assign hereafter the name *Typhode Fever*, to avoid the epithet *typhoid*, which indicates only an analogy of form, and *typhus* which excites alarm. But since *typhode fevor* is essentially contagious, the practical consequence ought to be, that physicians, the public and the administration, should in future, in sporadic cases, and especially in epidemics, adopt those hygienic and prophylactic measures that are generally indicated in contagious diseases."

